# Modeling the Origin and Possible Control of the Wealth Inequality Surge

**DOI:** 10.1371/journal.pone.0130181

**Published:** 2015-06-24

**Authors:** Yonatan Berman, Yoash Shapira, Eshel Ben-Jacob

**Affiliations:** School of Physics and Astronomy, The Raymond and Beverly Sackler Faculty of Exact Sciences, Tel-Aviv University, Tel-Aviv, Israel; University of Bristol, UNITED KINGDOM

## Abstract

The rapid increase of wealth inequality in the past few decades is a most disturbing social and economic issue of our time. In order to control, and even reverse that surge, its origin and underlying mechanisms should be revealed. One of the challenges in studying these mechanisms is to incorporate realistic individual dynamics in the population level in a self-consistent manner. Our theoretical approach meets the challenge by using interacting multi-agent master-equations to model the dynamics of wealth inequality. The model is solved using stochastic multi-agent iterated maps. Taking into account growth rate, return on capital, private savings and economic mobility, we were able to capture the historical dynamics of wealth inequality in the United States during the course of the 20th century. We show that the fraction of capital income in the national income and the fraction of private savings are the critical factors that govern the wealth inequality dynamics. In addition, we found that economic mobility plays a crucial role in wealth accumulation. Notably, we found that the major decrease in private savings since the 1980s could be associated primarily with the recent surge in wealth inequality and if nothing changes in this respect we predict further increase in wealth inequality in the future. However, the 2007–08 financial crisis brought an opportunity to restrain the wealth inequality surge by increasing private savings. If this trend continues, it may lead to prevention, and even reversing, of the ongoing inequality surge.

## Introduction

**Fig 1:**
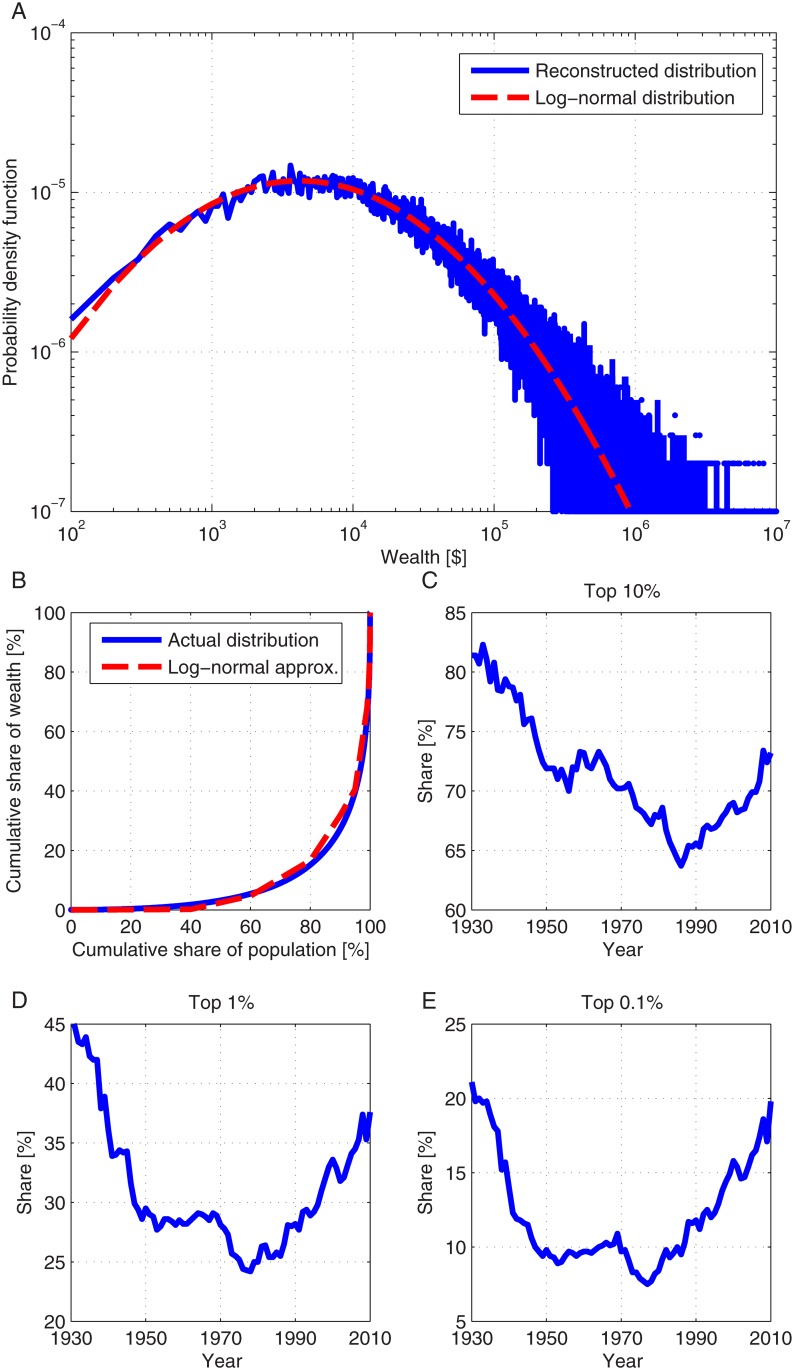
The distribution of wealth in the United States 1930–2010. A: The probability density reconstructed from the compiled historical data for 2007 (blue) and the probability density function of the approximated log-normal distribution (*μ* = 11.4 and *σ* = 1.75) (dashed red); B: The Lorenz curve of the wealth distribution for compiled historical data (blue) and for an approximated log-normal distribution (dashed red). The log-normal distribution parameters used were *μ* = 11.4 and *σ* = 1.75; C-E: The share of wealth owned by the top 10% of the population (C), the top 1% (D) and the top 0.1% (E). The presented data are by the courtesy of Gabriel Zucman [22, 23].

The surge in wealth inequality is one of the most disturbing social and economic issues of our time. The rapid increase in wealth inequality, mainly in western economies, has generated much effort to understand the origin and possible control of this trend [1–7]. For a comprehensive review of historical theories and analyses of wealth inequality please refer to Piketty [1]. Wealth inequality is generally thought to impose instabilities on economies and on the social structure of countries [1, 8–12]. Quantification of wealth inequality dynamics during the period 1930–2010 is depicted in Fig 1. Using the data for the share of wealth owned by different fractiles of the population, it is also possible to reconstruct the distribution of wealth within the population, presented in Fig 1. The Lorenz curve associated with this distribution and a theoretical approximation to a log-normal distribution are also presented in Fig 1. These results are consistent with previously known analyses of the wealth distribution. Other distributions, such as the Pareto and the stable Lévy, describe well the distribution of the high levels of wealth [13, 14]. However, the log-normal distribution is a better approximation of the lower wealth values [15–19].

A wide variety of factors have been proposed as effectors determining the wealth inequality dynamics, among them the changes in social structure and culture [20], the dramatic historical events of the 20th century [1], the basic essence of the capitalistic system [1, 11] and others suggested by historians and social scientists. Economists suggested other factors, such as taxation [1, 21–23], the democratization of education [24], technological advances and their effect on labor [25, 26], the rise of the financial sector [27, 28] and the dynamics of private savings [29–31]. In Piketty’s monumental work [1], he presents various mechanisms that contribute to the convergence and divergence of wealth inequality. His work also predicts that as long as the nominal return on capital investments (*r*) substantially overcomes the growth rate (*g*), the wealth inequality is expected to continue to increase, which may lead to social catastrophe if no measures are taken in order to reverse this trend.

The rationale behind the proposed factors and underlying mechanisms of the wealth inequality surge is generally accepted. However, a quantitative analysis is needed in order to understand the ways by which the different factors affect the wealth inequality dynamics and compare between the effects of the various factors. In simple words, we need to identify which of the factors play a major role and which a secondary role. Such an analysis will provide necessary information for policy making aiming to immediately suppress the soaring rate in wealth inequality and even further reduce it in the future.

Agent-based or individual-driven models for the dynamics of wealth accumulation have been recently studied, though not relating their results directly to wealth inequality. Other models generally ignore the strong interaction between agents within the population when addressing inequality [25, 29, 32]. Another model type incorporates wealth exchange between individuals in order to provide theoretical insights on the shape of the wealth distribution [16, 18, 19, 33–36]. Other models emphasize the importance of intergenerational elasticity and inheritance [29, 37, 38].

Here, we present a new approach of agent-based master equation devised to study the dynamics of the wealth inequality. The master equation describing the multi-agent dynamics is solved using stochastic multi-agent iterated maps. We incorporate in the model published information regarding the return on capital, growth rate, private savings and economic mobility as the parameters that govern these dynamics. The new framework allows us to bridge between the individual and population levels in a self-consistent manner. It also provides a theoretical framework to quantify the contributions of the factors affecting the wealth inequality dynamics and offers a valuable test-bed for predicting the effect of various policies on wealth inequality.

By implementing the modeling framework using a numerical simulation and considering the historical data for the different parameters in the US economy from 1930 to 2010, we compared and found an excellent agreement between the model and the historical dynamics of wealth inequality over this long period of time. As mentioned above, the new theoretical framework can be used as a predictor too, thus investigating the relative effect of the various parameters on future wealth inequality. It can be used to test which of the mechanisms that govern the dynamics of wealth inequality is primarily associated with the recent surge. We were also able to provide insights on the processes which might lead to the reduction of wealth inequality.

We point out the important distinction between income inequality and wealth inequality. Income inequality is usually easier to measure, and is the subject of the majority of the work published regarding economic inequality. In this work we study the dynamics of wealth inequality and not of income inequality. The two notions are closely related and sometimes very correlative. However, this is not always the case. In Denmark, for instance, the income inequality is one of the lowest in the world, while the level of wealth inequality is one of the highest in the world [39, 40]. For additional data regarding wealth inequality in different countries please refer to S1 Fig in the SI.

## The Modeling Framework

As mentioned earlier, we devised an agent-based master equation model for the wealth inequality dynamics. This master equation is presented by a set of stochastic differential equations of the individual wealth at discrete times. Hence, the model can be viewed as a set of coupled stochastic iterated maps of the individual wealth. Each equation incorporates a term describing the time change and propagation (between agents) of the wealth and labor income of individuals within the population. The individuals are characterized by two internal variables—their corresponding wealth and income deciles that govern their wealth dynamics. Furthermore, these internal variables themselves change in time for each individual, following the population collective dynamics and the individual dynamics. Therefore, the interaction between individuals within the population comes into play via those variables and their dynamics, as is detailed further below. In addition, we consider the processes of wealth gained by capital and by saving labor income and rent income for each individual. We also assume that labor income increases/decreases generally following the economic growth rate [1, 32] and incorporate economic mobility into the dynamics of labor income.

In short, the model describes the accumulation of wealth gained by individuals from capital and labor income, attenuated by spending. The labor income itself changes in time and is shuffled within the population through the economic mobility. For details regarding the model implementation, please refer to the Methods section.

More specifically, we formulate a stochastic iterated map for the wealth of the coupled individuals. The iterated map of the wealth dynamics of agent (*i*) at time *n*, *W*_*i*_(*n*), owned by each is given by:

$$\begin{array}{ccc} & & W_{i}(n+1)=W_{i}(n)+\\ & & \Gamma [n,\chi_{L_{i}(n)}][L_{i}(n)+\eta (n)W_{i}(n)]+\\ & & R[n,\chi_{W_{i}(n)}][1-\eta (n)]\kappa (n)W_{i}(n)\;. \end{array}$$ (1)

Eq (1) describes the accumulation of wealth for each individual (*i*) between time *n* and time *n*+1. In each iteration, income from savings (the second term in the right hand side) and income from the return on wealth (or the capital income that is the third term in the right hand side) are added to the wealth calculated in the previous iteration *W*_*i*_(*n*).

The savings term represents two sources of savings—stochastic labor income [*L*_*i*_(*n*)] and rent income [*η*(*n*)*W*_*i*_(*n*)]. The total amount of savings is given by a fraction of the sum of these sources. This fraction is denoted Γ[*n*, *χ*_*L*_*i*_(*n*)_], and is the product of two functions, for which the historical values are given: the relative savings fraction from the total income dependence on decile, Θ_Γ_[*χ*_*L*_*i*_(*n*)_], and the savings fraction from the disposable income, Γ_0_(*n*) [41–43].

**Fig 2:**
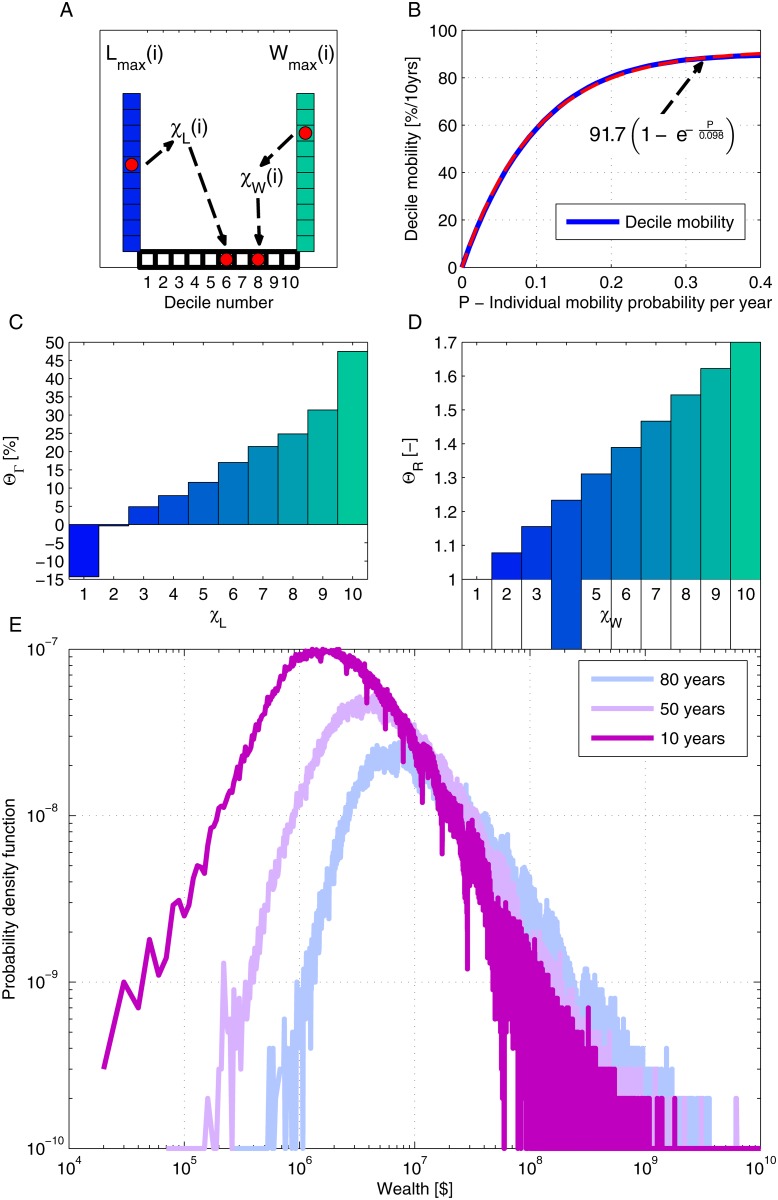
The characteristic behavior of the stochastic model. A: An illustration of the division to deciles—every time step *i*, each individual, with labor income *L* and wealth *W* is attached to a certain decile in labor income (*χ*_*L*_) and wealth (*χ*_*W*_) according to the distribution of the entire population; B: The decile mobility for 10 years is calculated following equation Eq (2) with *P* ranging from 0 to 0.4 per year. The decile mobility in the US was 75%–80% for 10-year periods during the past 50 years [44], leading to *P* values of 0.15–0.2 per year. The dashed red curve demonstrates that the decile mobility exponentially increases with *P*; C: The dependence of Θ_Γ_ on the labor income decile [41–43]; D: The dependence of Θ_*R*_ on the wealth decile; E: The model results for the distribution of wealth in the United States. The results were calculated for the historical values of the model parameters in the period 1930–2010, after 10 years (purple), 50 years (pink) and 80 years (light blue).

The disposable income fraction depends on the labor income. This dependence is introduced through the labor income decile, *χ*_*L*_*i*_(*n*)_, as illustrated in Fig 2. The rent income is a fraction of the wealth, denoted *η*(*n*)*W*_*i*_(*n*), where *η*(*n*), the rent fraction, is taken from historical data [23].

The stochasticity of the labor income is incorporated in the iterated map as follows: The labor income is assumed to increase or decrease generally following the economic growth [1, 32]. In addition, due to economic mobility, the income of an individual can significantly change with some probability, denoted by *P*. The dynamics of the stochastic labor income *L*_*i*_(*n*) owned by each individual (*i*) at time step [*n*−(*n*+1)] are given by:

$$\begin{array}{c} L_{i}(n+1)=\{\begin{array}{cc} \tilde{L}\cdot \prod_{j=1}^{n}[1+\tilde{g}(j)] & \text{with}\;\text{probability}\;P\\ L_{i}(n)\cdot [1+\tilde{g}(n)] & \text{otherwise}, \end{array} \end{array}$$ (2)

where $\tilde{L}$ is a value randomly chosen from the distribution used for the initial values of the population labor income. This distribution was taken from the actual income distribution in the US as presented in [22, 23].

The effective growth rate of the labor income $\tilde{g}\left(n\right)$ is dependent on the gross domestic product (GDP) real growth rate and is calculated using the historical data for the GDP growth rate and the fraction of wealth income from the total national income, that is termed *α*[1, 23]. This ensures the labor income proper propagation in time. For details regarding the calculation of $\tilde{g}$, please refer to the Methods section.

The decile mobility of a population is defined by the fraction of individuals that change their labor income decile within a given time interval [44]. Fig 2 depicts the dependence of the decile mobility on the value of the mobility probability *P* for an interval of 10 years. This way, the value of *P* is derived using the historical data for the decile mobility [44].

The capital income term in Eq (1) (the third term in the right hand side) represents the return earned from the individual’s investments and holdings, subtracted by the rent fraction. The return rate, *R*[*n*, *χ*_*W*_*i*_(*n*)_], is the product of two functions: the return dependence on wealth decile, Θ_*R*_[*χ*_*W*_*i*_(*n*)_], and the nominal rate of return, *R*_0_(*n*), for which the historical data are given [23].

**Fig 3:**
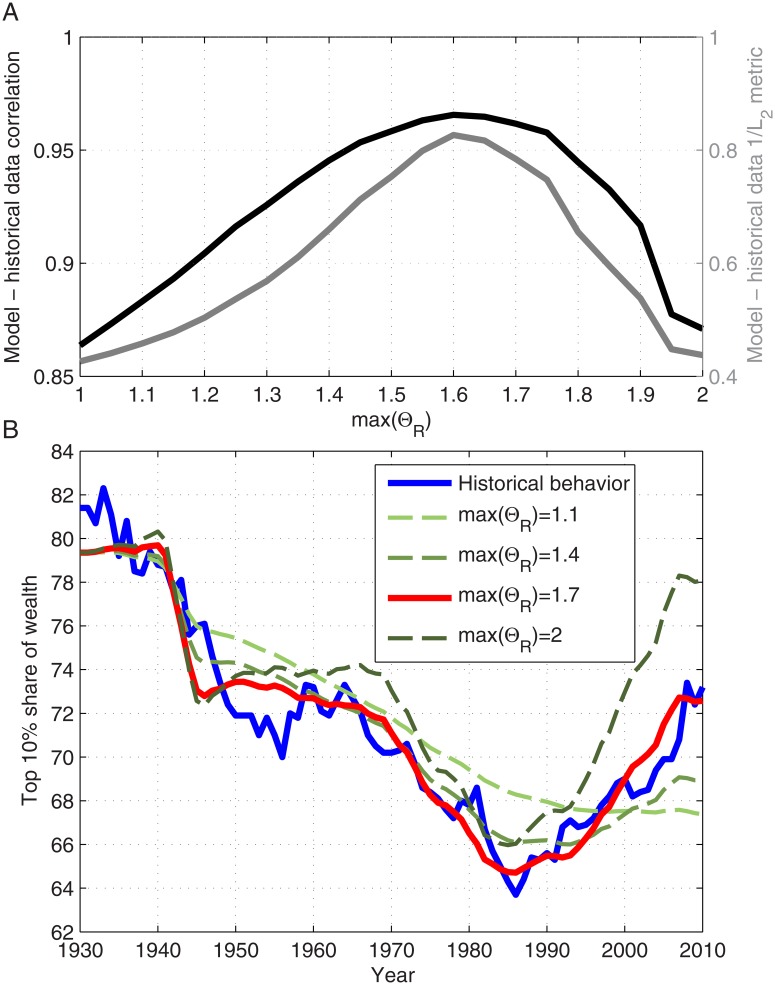
The dependence of the model results on *max* (Θ_*R*_). A: The dependence of the correlation between the model results and the historical data on *max* (Θ_*R*_) (black). Also presented is the dependence of 1/*L*_2_ metric between the model results and historical data on *max* (Θ_*R*_) (dark grey); B: The model results for the top 10% share of wealth in the United States during 1930–2010 for different values of *max* (Θ_*R*_). The results produced by implementing the described model were calculated using the historical data for the various parameters [22, 23]. The historical data (blue) were taken from Saez and Zucman [22].

A typical dependence of Θ_*R*_[*χ*_*W*_*i*_(*n*)_] on *χ*_*W*_*i*_(*n*)_ is depicted in Fig 2. We assume a linear dependence and set *max*(Θ_*R*_) (or Θ_*R*_(10)) to be 1.7. In order to properly estimate this value, the model was implemented, taking into account the historical values of each parameter for different values of *max*(Θ_*R*_). The results of this parameter estimation are presented in Fig 3.

In the case for which Θ_*R*_[*χ*_*W*_*i*_(*n*)_] is constant, no increase in wealth inequality can be possible, based on capital accumulation. Given that the return rate is the same for all deciles, the share of wealth owned by the top deciles will not increase and will only decrease or stay unchanged. The positive correlation between Θ_*R*_[*χ*_*W*_*i*_(*n*)_] and *χ*_*W*_*i*_(*n*)_ is originated by better terms of investment and more profitable investment possibilities accessible to rich individuals, but inaccessible to the poor. In addition, taking large risks, which is usually accompanied by a potential to a larger profit, is easier for the rich [1, 11, 45, 46].

We assume that all wealth deciles generally receive capital income. In practice, the lowest deciles might have negligible capital income. However, even if we neglect the capital income of the lowest wealth deciles, the results of the model change insignificantly. In order to test this argument, we performed several runs of the model in which we did not consider any capital income received by the lowest two wealth deciles. It was found that the results changed in less than 0.1% on average. Therefore, we continue with the dependence of Θ_*R*_[*χ*_*W*_*i*_(*n*)_] as presented, for the calculations to come.

The distribution of wealth in the population is used to determine the initial value of wealth for each individual. As previously observed (see Fig 1), it approximately follows a log-normal distribution. Fig 2 depicts the resulting wealth distribution for several time periods. The general log-normal behavior is conserved. In addition, since wealth and labor income originate from different distributions, they should be normalized. A normalization factor, *κ*(*n*) multiplies the contribution of income originated from wealth under the constraint that the historical values of the fraction of wealth income from the total national income (*α*) are maintained. For details regarding the calculation of the normalization factor, please refer to the Methods section.

The model enables to estimate the sensitivity of wealth inequality dynamics to each of the model parameters. It was found that on average, *V*_*α*_ = 0.6, meaning that a relative change of 1% in *α* is likely to result in an increase of approximately 0.6% in the wealth inequality. Additionally, *V*_Γ_0__ = −0.4, *V*_*η*_ = −0.1 and *V*_*g*_ = 5⋅10^−4^. These results indicate that *α*, the fraction of capital income from national income, and Γ_0_, the private savings fraction, are the most significant factors that determine the dynamics of wealth inequality. For details regarding the sensitivity calculation, please refer to the Methods section.

## Results

### Comparison with historical data

**Fig 4:**
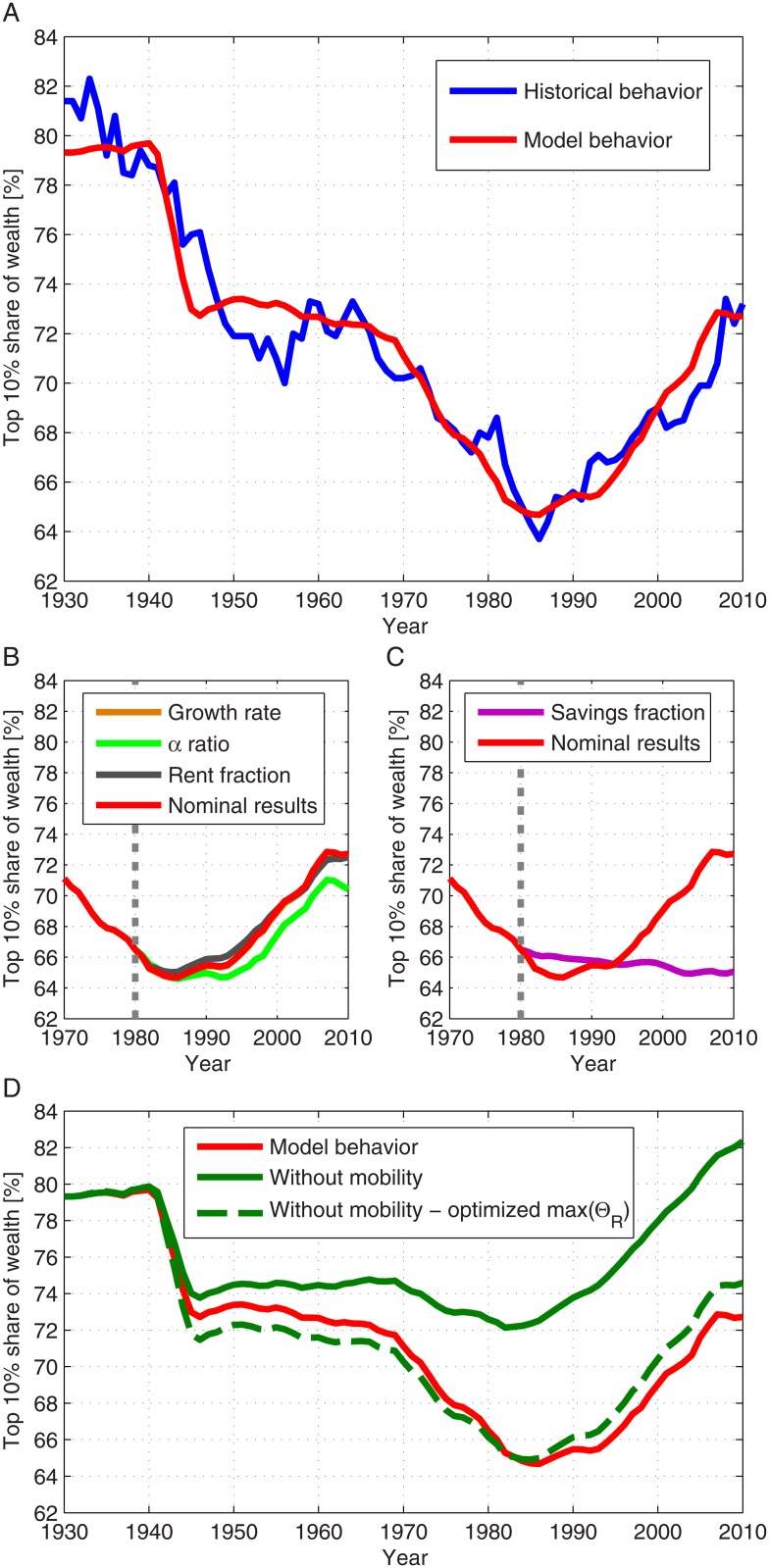
The model results for the top 10% share of wealth in the United States during 1930–2010. A: The results produced by implementing the described model were calculated using the historical data for the various parameters [22, 23]. These results are presented with economic mobility (*P* = 0.18 per year) (red). The historical data (blue) were taken from Saez and Zucman [22]; B-C: The top 10% share of wealth in the United States with averaged parameters during 1980–2010. The calculations were done for nominal parameters (red) and for each parameter, with its values during 1980–2010 taken as their average during 1930–1980, while the other parameters were taken with their nominal historical values. In B—average growth rate (orange), average *α* (light green) and average rent income fraction (gray). In C—average savings fraction (magenta). The dotted gray line separates the calculation using the nominal parameter values and the averaged parameter value; D: The model results for the top 10% share of wealth for nominal parameters (*P* = 0.18 per year) (red) and without economic mobility (*P* = 0 per year) (dark green). Also presented are the model results without economic mobility and optimized value of *max* (Θ_*R*_) (dashed dark green). In the presented calculation *max* (Θ_*R*_) = 1.3.

A comparison between the model results and 8 decades of the wealth inequality dynamics in the US is shown in Fig 4. More specifically, we show a comparison with wealth owned by the top decile in the US in the period 1930–2010 [22]. The results indicate the existence of a very high correlation (*ρ* = 0.96) between the modeled simulations and the historical data. This agreement provides an important test regarding the predictive power of the model. Notably, using the model we found that the economic mobility plays an essential role in governing wealth inequality dynamics. More specifically, when economic mobility is not included in the model, the model-market correlation is *ρ* = 0.61 vs. *ρ* = 0.96 in the case that this effector is included (Fig 4).

The effect of economic mobility is more significant when the economy becomes more dominated by the capital income. When *max*(Θ_*R*_) decreases, meaning that the effect of capital income is reduced, the correlation between the model and the market decreases as well. We considered a calculation in which the economic mobility was removed (*P* = 0), while changing the value of *max*(Θ_*R*_) in order to optimize the results when compared to the actual data. The optimal correlation achieved this way was *ρ* = 0.86, for *max*(Θ_*R*_) = 1.3 (see Fig 4).

The results in Fig 2 show that the dependence of decile mobility on the individual mobility probability *P* approximately follows $91.7\left(1-e^{-\frac{P}{0.098}}\right)$ and reaches an asymptotic value. Therefore, the sensitivity of the model results to the value of the individual mobility probability is found to be relatively low for *P* > 0.05. Within the interval 0.05 < *P* < 0.2 per year (the nominal value is 0.18), the correlation between the modeled top decile share of wealth and the historical data was 0.94 < *ρ* < 0.96. This indicates that a further increase of mobility is likely to have a small effect on wealth inequality. Notably, in the current state of decile mobility in the US, the wealth inequality is almost independent on plausible changes in mobility. This low sensitivity to the mobility probability is also depicted in S2 Fig in the SI.

The good agreement between the model results and the historical data motivated us to take the analysis a step further and to use the model for two main purposes:

1. Understanding the mechanisms that drive the inequality increase and decrease, particularly indicating which mechanism is associated with the recent surge in inequality.
2. Making predictions for the future, and specifically determining what set of circumstances might lead to the reduction of wealth inequality or to its further increase.

### The driving mechanisms of wealth inequality

Evident from Fig 1 and a well-known fact is that during the 20th century the wealth inequality in the US and in most western countries had significantly decreased, mostly during 1940–1970, and then increased since 1980. The results presented in Fig 4, and the calculated sensitivity values provide an intuition for the mechanisms of wealth inequality decrease and increase. First, it can be concluded that in general, the wealth inequality tends to decrease as long as the inequality of labor income is lower than the inequality of wealth and the fraction of wealth income from the total national income (*α*) is less than 50%. As the savings fraction increases this trend is accelerated. This functions as the main mechanism which contributes to the decline in wealth inequality during the period 1930–1980. High growth rates, relatively small *α* values and a relatively large savings fraction are responsible for the acceleration of the decline for certain years, most notably following the Second World War. During the period 1980–2010, growth rates had declined, the fraction *α* had become slightly larger than before, but most significantly, the savings fraction had dramatically decreased. From 1980 onward, capital income became dominant, whereas before, labor income was dominant. Though the labor income was still larger than capital income, the savings fraction had fallen significantly, so that effectively, capital income was substantially larger than labor income, a condition that naturally causes an increase of wealth inequality.

**Fig 5:**
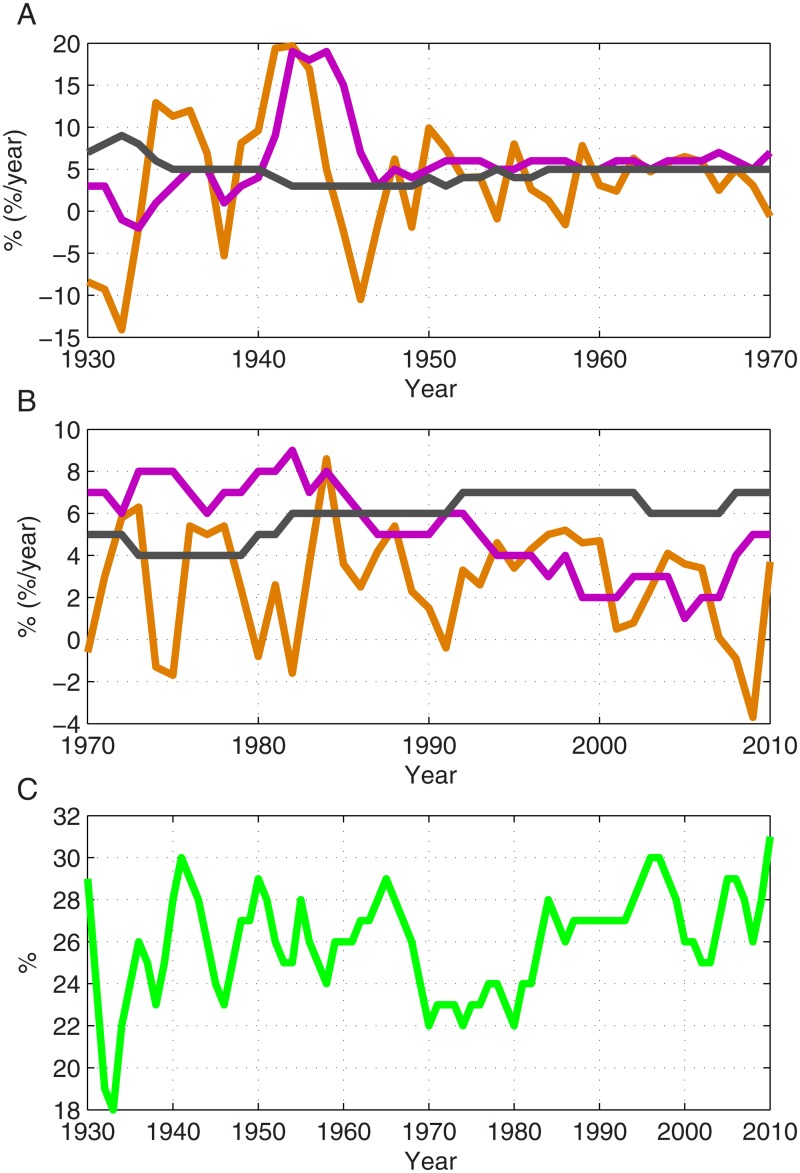
The historical values of the model parameters 1930–2010. The data are presented for the GDP growth rate (orange), private savings fraction (magenta) and rent income fraction (gray) for the periods 1930–1970 (A) and 1970–2010 (B). In addition, the historical values of the *α* fraction (light green) are presented (C). The data were taken from Saez and Zucman [22].

In order to support these arguments, we performed several numerical simulations. In each simulation, one of the parameters was taken as equal to its average during the period 1930–1980 in the period 1980–2010. These results, presented in Fig 4, confirm our hypothesis—that the changes in the growth rate during 1980–2010 have a negligible direct effect on the inequality. In addition, although the values of *α* had become larger during this period, it cannot explain the dramatic change in the course of inequality. The results demonstrate that the dramatic changes in the savings fraction had the most crucial role in changing the inequality trend in the period in discussion. The historical values of the different model parameters are presented in Fig 5 for the period 1930–2010 in the US.

### Assessment of the model’s predictive power

In order to test the predictive power of our model we performed retrospective predictions of the wealth inequality for two periods: (I) 1980–2010; (II) 2000–2010. The retrospective predictions were done as follows. First, for periods I and II we calculated the wealth inequality dynamics using the historical values of the different parameters (Fig 4). Second, we calculated the wealth inequality dynamics for periods I and II for four sets of test parameters (representing different policy scenarios) defined below:

1. Unchanged parameters—The values of the parameters are taken as the averaged value of the parameters during the preceding periods (1970–1980) and (1990–2000) for periods I and II, respectively.
2. Decreasing savings scenario—The values of the parameters are taken as described in (1), except for the savings fraction that linearly decreases to 1% at 2010.
3. Decreasing savings, increasing *α* scenario—The values of the parameters are taken as described in (1), except for the savings fraction that was taken to be linearly decreasing to 1% at 2010 and the fraction *α* that was taken to be linearly increasing to 32% at 2010.
4. Increasing savings, decreasing *α* scenario—The values of the parameters are taken as described in (1), except for the savings fraction that linearly increases to 15% at 2010 and the fraction *α* that linearly decreases to 15% at 2010.

**Fig 6:**
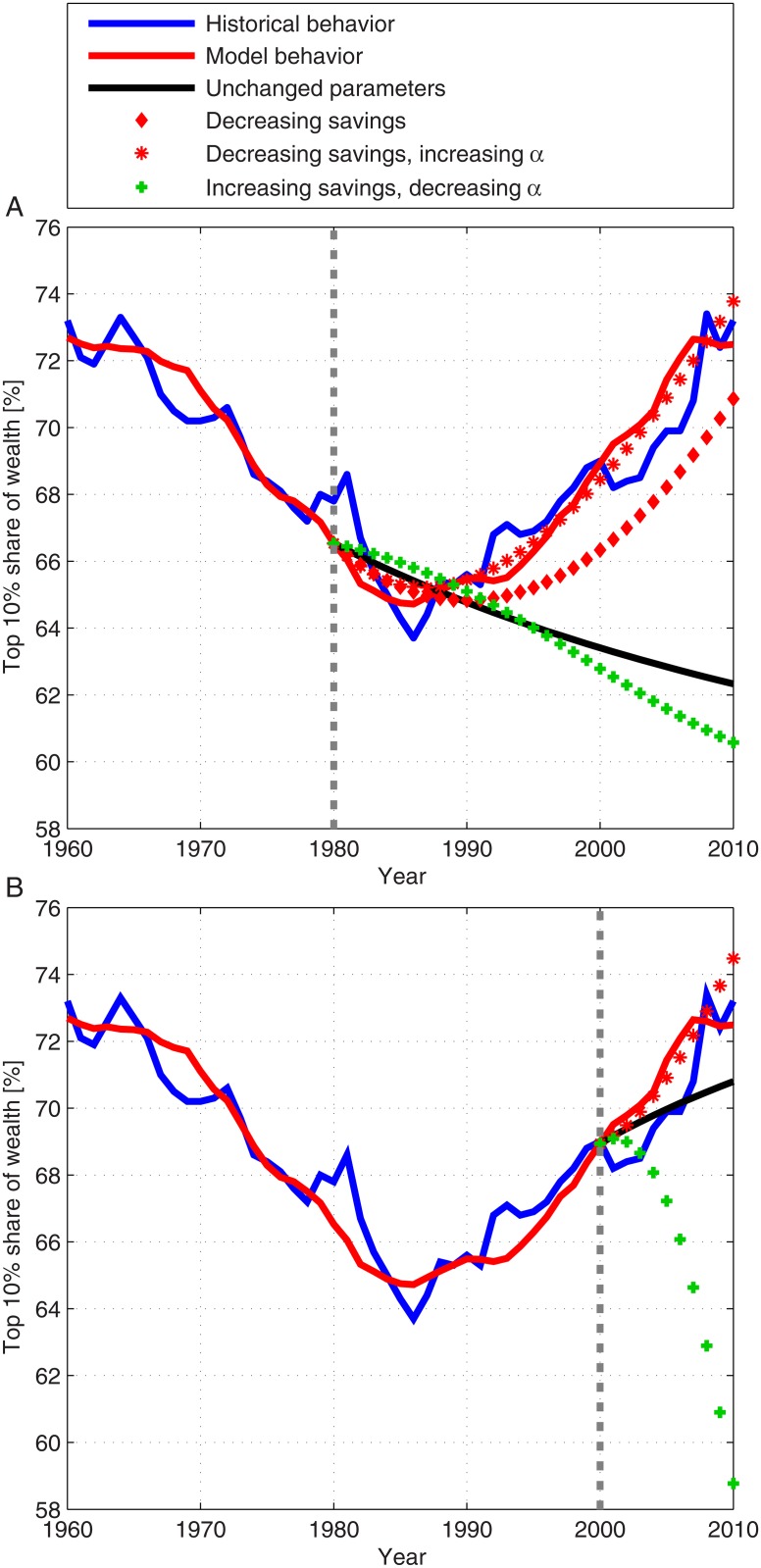
Retrospective predictions of the wealth inequality in the US. The blue and red curves present the historical market behavior and the model behavior for the historical values of the parameters, respectively (see Fig 4). The results for the various scenarios during 1980–2010 (A) and during 2000–2010 (B) are also presented: Unchanged parameter scenario (solid black curve), decreasing savings scenario (red diamonds), decreasing savings and increasing *α* scenario (red stars) and increasing savings and decreasing *α* scenario (green crosses). The significant difference between the results for the increasing savings and decreasing *α* scenario in (A) and (B) is due to the fast savings fraction increase in (B) (from 4% to 15% within 10 years), compared to a mild increase in (A) (from 7% to 15% within 30 years). The dotted gray line separates the calculation using historical parameter values and the retrospective prediction.

The results of these retrospective predictions are presented in Fig 6. The results of such retrospective predictions for additional scenarios are presented in S3 Fig in the SI. These results illustrate the predictive power of the new framework. These results imply that the model may be used to test the effect of future financial policies, as we describe in the next section.

### Estimation of future wealth inequality

Using the modeling framework, and given its reliability, we proceed to make predictions of the future wealth inequality dynamics in the US. Making such predictions rely, however, on making assumptions on future policy as reflected by the corresponding future model parameters. So we predicted wealth inequality dynamics for 2010–2030 for the following scenarios:

1. Nominal constant parameter scenario—The values of the parameters are taken as constant during 2010–2030 and each is equal to its average during the period 2000–2010.
2. Extrapolated parameter scenario—The values of the parameters are linearly extrapolated during 2010–2030 based on their values during 2000–2010.
3. Increasing *α* scenario—The values of the parameters are taken as described in (1), except for the fraction *α* that linearly increases to 35% at 2030.
4. Increasing savings scenario—The values of the parameters are taken as described in (1), except for the savings fraction that linearly increases to 10% at 2030.

**Fig 7:**
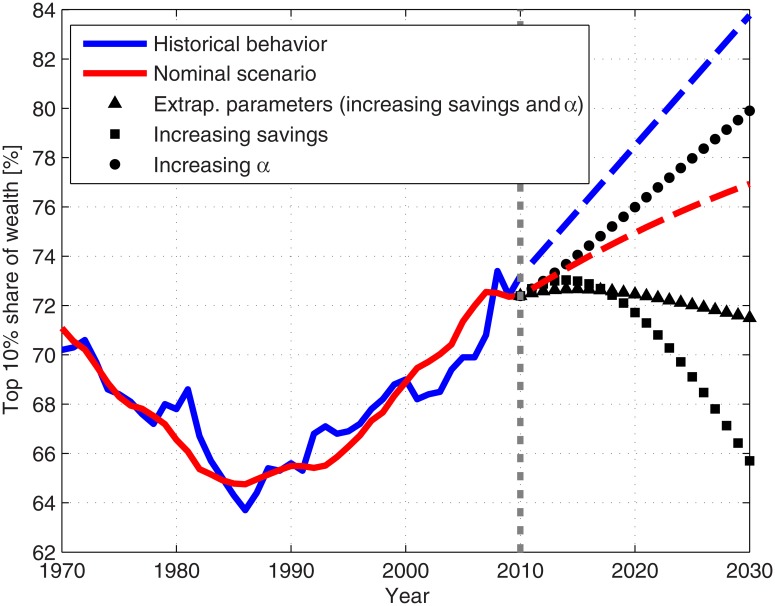
Predictions of future wealth inequality. The blue and red curves present the historical and model results, respectively (see Fig 4). The dashed curves display the predictions of the future inequality. The results for the various parameter scenarios during 2010–2030 are also presented: extrapolated parameter scenario (triangles), increasing savings scenario (squares) and increasing *α* scenario (circles). In addition, a linear extrapolation of the wealth inequality during 2000–2010 to 2010–2030 is presented as well (dashed blue curve). The dotted gray line separates the calculation using historical parameter values and the prediction for future inequality.

In addition, a simple linear extrapolation of the historical wealth inequality during 2000–2010 was computed for comparison. These various scenarios can be interpreted as the outcomes of regulation and policy, changes in social structure and various other economic, political and social developments. Such changes and developments are impossible to predict, and most forecasts are given for a year or two ahead. The probability for each of the various scenarios to occur is not predictable. However, they form a diversified span of possible future outcomes. The model results for the described parameter scenarios are presented in Fig 7. As expected, if the average of the recent parameter values is taken into account, the wealth inequality is likely to increase at a similar pace as in 2000–2010. However, when extrapolating the parameters, it remains almost constant during 2010–2030. This is due to the small increase in private savings fraction following the global financial crisis on one hand, and the increase of *α* on the other hand [23]. These two processes restrain one another’s effect. In addition, the results for the increasing *α* scenario and the increasing savings scenario demonstrate that these parameters have indeed a large effect on wealth inequality.

**Fig 8:**
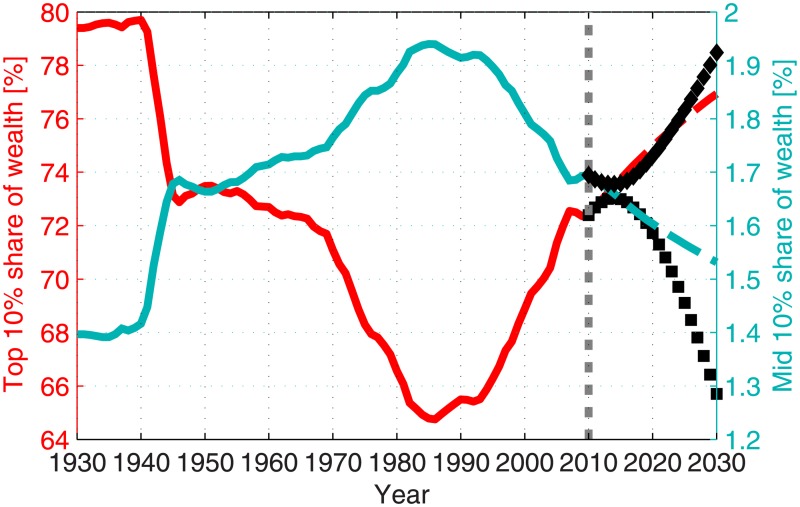
Prediction of the top and mid deciles shares of wealth. The solid red and cyan curves present the model results for the share of wealth owned by the top and mid deciles, respectively. These results were calculated using the historical parameter values. The dashed curves display the projections of the future share of wealth for the nominal constant parameter scenario. The projections of the future share of wealth for the increasing savings scenario are presented for the top 10% share (black squares) and for the mid 10% share (black diamonds). The dotted gray line separates the calculation using historical parameter values and the projection for future inequality.

A future increase in the wealth owned by the top decile is likely to be accompanied by a decrease in the share of wealth owned by lower deciles, and vice versa, as depicted in Fig 8. These results demonstrate that reducing the share of wealth owned by the top decile due to one or some of the mechanisms will clearly lower wealth inequality.

## Discussion

We devised a stochastic model for the dynamics of wealth inequality. Using the historical values to estimate the model parameters, we were able to reproduce the historical values of wealth inequality in the United States during 1930–2010. Based on the simulation results it can be concluded that within the framework of the parameters taken into account, the effect of the growth rate on the wealth inequality is minor. The private savings fraction and the fraction of capital income in the national income (*α*) were found to be highly influential on wealth inequality, and essentially dominate its dynamics. This conclusion makes it possible to identify the mechanisms that contribute to the increase and decrease of wealth inequality. It also indicates that in the early 1980s the United States economy gradually switched from being income-dominated [1, 11, 23] to being capital-dominated. In addition, the simulation results demonstrate that under plausible assumptions, the possibility of wealth inequality divergence within the next few decades can be reversed.

The obtained results imply that the wealth inequality surge in the United States since the 1980s is primarily due to the major decrease in private savings in the same period. The recent financial crisis, however, led to an increase in private savings back to levels characterizing the mid-1990s. This makes the crisis an opportunity to reduce inequality if this trend continues. In addition, the observed behaviors of wealth inequality as well as of private savings in the past few decades are almost universal. Wealth inequality in most developed countries is gradually increasing since the 1980s [1]. This increase is accompanied by a decrease in private savings, especially in the US and Japan [1, 23, 47]. For more information and data please refer to S1 Fig in the SI.

The measure of wealth inequality that was used in the above-mentioned simulations is the wealth share owned by the top decile. Different measures can be also used. The Gini coefficient, for example, produces very similar results to those presented for the wealth share of the top decile, with a very high correlation between the historical data and the model results. However, when the top 0.1% or 0.01% shares of wealth are taken into account, the results diverge from the historical data. Among those top fractiles, capital income is dominant and the fraction *α* becomes effectively much larger than 50%, which substantially change the dynamics of wealth accumulation. Moreover, the access to high return investments [1, 11, 45] and the alleged ability to effect regulation due to wealth [1, 11], naturally contribute to a growing gap even within the top 10%, 1% or 0.1% [14]. For more information and data please refer to S4 Fig in the SI. We also note that while economic mobility is usually measured and discussed in terms of labor income, it can be also interpreted in terms of wealth, via the introduction of systemic risk [48]. Systemic risk is likely to have a larger effect within the top fractions of the population compared to its effect within deciles. This is due to the vulnerability of the financial sector to systemic risk relative to other sectors in the economy [48–50]. In addition to systemic risk, other mechanisms might be successful in explaining the extreme inequality within the top fractiles. Such an additional mechanism might be preferential attachment, in a way similar to the mechanism presented for network growth or citation networks [51, 52]. This mechanism is sometimes associated with processes characterized by “the rich get richer” behaviors. Such processes are also known to result in a Pareto tail [13, 14].

The observed results also hint how taxation could be addressed. Income and consumption taxes effectively reduce the savings fraction and therefore are expected to increase wealth inequality. On the other hand, progressive income tax also reduces the labor income inequality, and therefore the total effect of such taxation on the wealth inequality cannot be simply determined. Capital gains taxes effectively reduce *α* and therefore are expected to reduce wealth inequality. In addition, taxation is generally thought to reduce growth rates [53, 54], although different perspectives can also be found [55]. Judging by the results that imply a relatively weak effect of the growth rate on wealth inequality, no significant second-order effects on the inequality are expected due to the interplay between growth and taxation. A thorough investigation on the effects of taxation on inequality, using the model, is left for future work.

Reliable modeling of wealth inequality dynamics with models such as this one can serve regulators and policy makers in their efforts to reduce wealth inequality. However, while describing the mechanisms that might contribute to lowering wealth inequality, such as increasing private savings fraction and eliminating the dominance of capital income over effective labor income, the implications of such processes on other aspects of the economy were not considered. Such implications should be taken into account when policy and regulation are considered. Notably, substantially increasing private savings might have a major positive effect on wealth inequality. However, its marginal effect is reduced as the savings rate increases and a very high rate might also limit the GDP growth [54, 56]. Therefore, optimization between maximizing product growth and controlling wealth inequality can be achieved, leading to the formation of a more successful economy.

More research should be carried out to establish the above: further validation of the model by considering additional economies; thorough investigation of the effects of taxation on wealth inequality; incorporating additional factors in the model and quantitative estimation of the parameter values leading to the decrease of wealth inequality.

## Methods

### Data retrieval

We used the historical values of several economic properties for the presented model—the historical growth rate, savings fraction and the fraction of capital income from national income in the United States during 1930–2010. These data, along with the data for the wealth share owned by the top decile are by the courtesy of Gabriel Zucman [22, 23]. The data used for the calculating the proper value of the mobility probability *P*, were taken from [44]. The dependence of the savings on income decile, taking into account both the disposable income and the relative saving fraction of each decile originates in [41–43].

### The implementation of the individual-based stochastic model

The presented model was implemented by a numerical simulation. Each individual was given an initial wealth and an initial annual income, randomly distributed according the known distributions for the United States [22, 57]. The initial value distributions are important, since the large difference between the inequality of wealth and income is an important factor in the dynamics of wealth inequality. Following Eqs (1–2), at every time step, equivalent to one year, the wealth and labor income of each individual were updated. As previously stated, the values wealth and income were normalized so that the given value of *α* was exercised. In addition, following the update of the labor income of the whole population, the values within each decile were multiplied by a factor close to 1, so that the historical inequality value of labor income was maintained.

Following the propagation of wealth and labor income of each individual, the division of the population to deciles in income and wealth was done. An important detail is that the time propagation was done for the whole population and not for each individual separately. Otherwise, the division to deciles could not have been properly done, distorting the simulation results. Following the division to deciles, the share of wealth owned by the top wealth decile, was calculated. The described procedure was done for each time step until the end of the available data.

Due to the randomly distributed initial values of wealth and income, and due to the stochastic nature of the implemented economic mobility, each run of the simulation produces slightly different results, given the same parameters. Therefore, a sufficient population size should be taken in order to obtain consistent results. We used a population of *N* = 10^7^ individuals, which provided an average difference of less than 0.1% in wealth inequality between two independent runs of the simulation.

### $\tilde{g}$ calculation

In order to calculate the labor income effective growth rate $\tilde{g}$, let us denote the GDP real growth rate as *g*(*n*), the total national income as *T*(*n*), the total income gained from wealth as *r*(*n*)*W*(*n*) and the total labor income by *L*(*n*). We obtain 4 simple relations:

$$\begin{array}{ccc} & & r(n)W(n)+L(n)=T(n)\\ & & r(n+1)W(n+1)+L(n+1)=T(n+1)\\ & & T(n+1)/T(n)=1+g(n)\\ & & L(n+1)/L(n)=1+\tilde{g}(n)\;. \end{array}$$ (3)

In addition, given that $\alpha \left(n\right)=\frac{r\left(n\right)W\left(n\right)}{r\left(n\right)W\left(n\right)+L\left(n\right)}$, we obtain the relation $\frac{r\left(n\right)W\left(n\right)}{L\left(n\right)}+1=\frac{T\left(n\right)}{L\left(n\right)}=\frac{1}{1-\alpha \left(n\right)}$. Considering the relations in 3, we now get $\frac{1+g\left(n\right)}{1+\tilde{g}\left(n\right)}=\frac{1-\alpha \left(n\right)}{1-\alpha \left(n+1\right)}$. Finally, the labor income effective growth rate $\tilde{g}$ is obtained:

$$\begin{array}{c} \tilde{g}(n)=(1+g(n))\frac{1-\alpha (n+1)}{1-\alpha (n)}-1\;. \end{array}$$ (4)

The value of $\tilde{g}$ is very close to the value of the real growth rate, as the change in *α* between consecutive years is mild.

### Normalization factor calculation

The distributions of income and wealth are properly normalized each time step of the model run. In order to perform this normalization, we use the factor *κ*(*n*) (see Eq (1)). The normalization is implemented under the constraint that the historical values of *α*, the fraction of capital income from national income are maintained. This way, together with the calculated value of $\tilde{g}$, the time propagation of the labor income is proper.

By definition, $\alpha \left(n\right)=\frac{R_{0}\left(n\right)\sum_{i}W_{i}\left(n\right)}{R_{0}\left(n\right)\sum_{i}W_{i}\left(n\right)+\sum_{i}L_{i}\left(n\right)}$. In addition, *β*(*n*), the ratio between total wealth and national income at time step *n* equals *α*(*n*)/*R*_0_(*n*) [1]. It follows that $\frac{\sum_{i}L_{i}\left(n\right)}{\sum_{i}W_{i}\left(n\right)}=\frac{1-\alpha \left(n\right)}{\beta \left(n\right)}$. Combining these relations together with Eq (1) we get the constraint:

$$\begin{array}{c} \kappa (n)=\frac{\beta (n)}{1-\alpha (n)}\frac{\sum_{i}L_{i}(n)}{\sum_{i}W_{i}(n)}\;. \end{array}$$ (5)

### Sensitivity calculation

Let us denote the model results for wealth inequality by *S*(*t*), measured by the wealth share of the top 10%. It is now possible to define the sensitivity of *S*(*t*) to each parameter *X*(*t*) for time *t* by: $V_{X}\left(t\right)=\frac{X\left(t\right)}{S\left(t\right)}\frac{\partial S\left(t\right)}{\partial X\left(t\right)}$. All parameters are found to have an effect on wealth inequality, but the magnitudes of these effects differ considerably. We found that on average, *V*_*α*_(*t*) = 0.6, meaning that a relative change of 1% in *α* is likely to result in an increase of approximately 0.6% in wealth inequality. Additionally, *V*_Γ_0__(*t*) = −0.4, *V*_*η*_(*t*) = −0.1 and *V*_*g*_(*t*) = 5⋅10^−4^.

## Supporting Information

S1 Fig Wealth inequality in different countries. A: The private savings fraction is presented for the United States (blue), France (light blue), Germany (yellow), Italy (green) and Japan (dashed red) for 1975–2010. The data is taken from [23]; B: The top 10% share of wealth is presented for the United States (blue curve) for 1930–2010, as well as for Denmark (red square), France (light blue circle), Germany (yellow circle), Italy (green circle) and Japan (red circle) in 2000. The data is taken from [40]. This figure displays once again the link between private savings and wealth inequality. In addition, it shows that low income inequality does not necessarily imply low wealth inequality, as demonstrated by Denmark. In Denmark, the income inequality is one of the lowest in the world, while the level of wealth inequality is one of the highest in the world [39, 40].
(PDF)

S2 Fig The correlation between wealth and labor income. The correlation is presented for 1970 (blue) and for 2010 (black) as a function of the mobility probability *P*. The dashed red curves demonstrate that the correlation is inversely proportional to the square of the mobility probability. The figure demonstrates that the sensitivity of this correlation to the value of *P* becomes very low at about *P* = 0.1.
(PDF)

S3 Fig Retrospective predictions of wealth inequality in the US. The blue and red curves present the historical market behavior and the model behavior for the historical values of the parameters, respectively (see Fig 4). The results for the various scenarios during 1980–2010 (A) and during 2000–2010 (B) are also presented: Unchanged parameter scenario (solid black curve), increasing savings scenario (green squares), decreasing savings scenario (red diamonds), increasing *α* scenario (red triangles), decreasing savings scenario (green circles), increasing savings and decreasing *α* scenario (green crosses) and decreasing savings and increasing *α* scenario (red stars). The dotted gray line separates the calculation using historical parameter values and the retrospective prediction.
(PDF)

S4 Fig Wealth inequality among top wealth fractiles in the United States 1930–2010. A-D: The historical share of wealth (blue) and the model results (red) owned by the top 10% of the population (A), the top 1% (B), the top 0.1% (C) and the top 0.01% (D). The historical data was taken from Saez and Zucman [22]. The results imply that different mechanisms govern the accumulation of wealth within the top fractiles of the population compared to within the vast majority of the population. Among the top fractiles, capital income is dominant and the fraction *α* becomes effectively much larger than 50%, which substantially change the dynamics of wealth accumulation. Moreover, the positive correlation between wealth and return on wealth [1, 11, 45] naturally contribute to a growing gap within the top 10%, 1% or 0.1%. We also note that the discrepancy between the model results and the data might be contributed to inaccurate data. Due to the smaller values, the data are more sensitive to errors. In addition, there exists a debate regarding the data provided by Saez and Zucman [22], which are inconsistent with other sources of wealth data [7].
(PDF)

## References

[1] Piketty T. Capital in The Twenty-First Century. Cambridge: Harvard University Press; 2014.

[2] Aghion P, Williamson JG. Growth, Inequality, and Globalization: Theory, History, and Policy. Cambridge: Cambridge University Press; 1998.

[3] Keister LA. Wealth in America: Trends in Wealth Inequality. Cambridge: Cambridge University Press; 2000.

[4] Piketty T, Saez E. Income inequality in the United States, 1913–1998 (series updated to 2000 available). National bureau of economic research. 2001; Working paper No. w8467.

[5] Sen A. On Economic Inequality. Oxford: Clarendon Press; 1973.

[6] Plog S, Heitman C. Hierarchy and social inequality in the American Southwest, AD 800–1200. Proc Natl Acad Sci USA. 2010; 107: 19619–19626. 10.1073/pnas.1014985107 21059921 PMC2993351

[7] Kopczuk W. What do we know about the evolution of top wealth shares in the United States?. J Econ Perspect. 2015; 29: 47–66. 10.1257/jep.29.1.47

[8] Huang ZF, Solomon S. Finite market size as a source of extreme wealth inequality and market instability. Physica A. 2001; 294: 503–513. 10.1016/S0378-4371(01)00113-3

[9] Louzoun Y, Solomon S, Goldenberg J, Mazursky D. World-size global markets lead to economic instability. Artif Life. 2003; 9: 357–370. 10.1162/106454603322694816 14761256

[10] Tavoni A, Dannenberg A, Kallis G, Löschel A. Inequality, communication, and the avoidance of disastrous climate change in a public goods game. Proc Natl Acad Sci USA. 2011; 108: 11825–11829. 10.1073/pnas.1102493108 21730154 PMC3141931

[11] Reich RB. Beyond Outrage: What Has Gone Wrong With Our Economy and Our Democracy and How to Fix it. New York: Random House; 2012.

[12] Stiglitz JE. The Price of Inequality: How Today’s Divided Society Endangers Our Future. New York: Norton, W. W. & Company, Inc; 2012.

[13] Montroll EW, Shlesinger MF. On 1/f noise and other distributions with long tails. Proc Natl Acad Sci USA. 1982; 79: 3380–3383. 10.1073/pnas.79.10.3380 16593191 PMC346419

[14] Nagayama F. Wealth Inequality Among the Forbes 400 and US Households Overall. Federal Reserve Bank of Philadelphia research department special report. 2013; Available: http://www.philadelphiafed.org/research-and-data/publications/research-rap/2013/wealth-inequality-among-forbes-400-and-us-households-overall.pdf.

[15] Benhabib J, Bisin A. The Distribution of Wealth: Intergenerational transmission and redistributive policies. 2007. NYU Working Paper; Available: www.econ.nyu.edu/user/bisina/ParetoDistribution2007.pdf.

[16] Chatterjee A, Chakrabarti BK. Kinetic exchange models for income and wealth distributions. EPJ B. 2007; 60: 135–149. 10.1140/epjb/e2007-00343-8

[17] Chatterjee A, Sinha S, Chakrabarti BK. Economic inequality: Is it natural? Curr Sci. 2007; 92: 1383–1389.

[18] Patriarca M, Chakraborti A. Kinetic exchange models: From molecular physics to social science. Am J Phys. 2013; 81: 618–623. 10.1119/1.4807852

[19] Jones CI. Pareto and Piketty: The macroeconomics of top income and wealth inequality. National bureau of economic research. 2014; Working paper No. w20742.

[20] Boudon R. Education, Opportunity, and Social Inequality: Changing Prospects in Western Society. New York: Wiley-Interscience; 1974.

[21] Avi-Yonah RS. Taxation and inequality. Challenge. 2014; 57: 97–104. 10.2753/0577-5132570305

[22] Saez E, Zucman G. Wealth inequality in the United States since 1913: Evidence from capitalized income tax data. National bureau of economic research. 2014; Working paper No. w20625.

[23] Piketty T, Zucman G. Capital is back: Wealth-income ratios in rich countries, 1700–2010. Q J Econ. 2014 10.1093/qje/qju018

[24] Stiglitz JE. Education and inequality. Ann Am Acad Polit Soc Sci. 1973; 409: 135–145. 10.1177/000271627340900115

[25] Heckman JJ, Lochner L, Taber C. Explaining rising wage inequality: Explorations with a dynamic general equilibrium model of labor earnings with heterogeneous agents. Rev Econ Dynam. 1998; 1: 1–58. 10.1006/redy.1997.0008

[26] Deaton A The Great Escape: Health, Wealth and the Origins of Inequality. Princeton: Princeton University Press; 2013.

[27] Johansen A, Sornette D. Modeling the stock market prior to large crashes. EPJ B. 1999; 9: 167–174. 10.1007/s100510050752

[28] Claessens S, Perotti E. Finance and inequality: Channels and evidence. J Comp Econ. 2007; 35: 748–773. 10.1016/j.jce.2007.07.002

[29] Cagetti M, De Nardi M. Wealth inequality: Data and models. Macroecon Dynam. 2008; 12: 285–313. 10.1017/S1365100507070150

[30] Alvarez-Cuadrado F, el Attar Vilalta M. Income inequality and saving. 2012. Working paper; Available: anon-ftp.iza.org/dp7083.pdf.

[31] Ng YK. Is an Increasing Capital Share under Capitalism Inevitable? Nanyang Technological University EGC Report No: 2014/10; Available: www3.ntu.edu.sg/hss2/egc/wp/2014/2014-10.pdf.

[32] Piketty T, Saez E. Inequality in the long run. Science. 2014; 344: 838–843. 10.1126/science.1251936 24855258

[33] Ispolatov S, Krapivsky PL, Redner S. Wealth distributions in asset exchange models. EPJ B. 1998; 2: 267–276. 10.1007/s100510050249

[34] Louzoun Y, Solomon S. Volatility driven market in a generalized Lotka–Voltera formalism. Physica A. 2001; 302: 220–233. 10.1016/S0378-4371(01)00466-6

[35] Cirillo P, Redig F, Ruszel W. Duality and stationary distributions of wealth distribution models. J Phys A. 2014; 47: 085203 10.1088/1751-8113/47/8/085203

[36] Bagatella-Flores N, Rodriguez-Achach M, Coronel-Brizio HF, Hernandez-Montoya AR. Wealth distribution of simple exchange models coupled with external dynamics. Physica A. 2015; 417: 168–175. 10.1016/j.physa.2014.07.081

[37] De Nardi M. Wealth inequality and intergenerational links. Rev Econ Stud. 2004; 71: 743–768. 10.1111/j.1467-937X.2004.00302.x

[38] Benhabib J, Bisin A, Zhu S. The distribution of wealth and fiscal policy in economies with finitely lived agents. Econometrica. 2011; 79: 123–157. 10.3982/ECTA8416

[39] Elgar FJ, Aitken N. Income inequality, trust and homicide in 33 countries. Eur J Pub Health. 2011; 21: 241–246. 10.1093/eurpub/ckq068 20525751

[40] Davies JB, Sandström S, Shorrocks A, Wolff EN. The Level and Distribution of Global Household Wealth. Econ J. 2011; 121: 223–254. 10.1111/j.1468-0297.2010.02391.x

[41] OECD StatExtracts Database. Available: sstats.oecd.org.

[42] Anderson JM. The wealth of US families: Analysis of recent Census Data. 1999. Available: www.nber.org/sipp/docs/wp233.pdf.

[43] Office for National Statistics. The effects of taxes and benefits on household income, 1977–2012/13. Available: www.ons.gov.uk/ons/dcp171780 368301.pdf.

[44] Bradbury K, Katz J. Trends in U.S. family income mobility, 1967–2004. Federal reserve bank of Boston. Working Paper; Available: www.bostonfed.org/economic/wp/wp2009/wp0907.pdf.

[45] Dalko V, Klein LR, Curme C, Kenett DY, Stanley HE, et al Income inequality and information monopoly in financial markets. Q J Econ. 2014; In press.

[46] Boyce JK. Let them eat risk? Wealth, rights and disaster vulnerability. Disasters. 2000; 24: 254–261. 10.1111/1467-7717.00146 11026158

[47] Ohtake F, Kohara M, Okuyama N, Yamada K. Growing inequalities and their impacts in Japan. GINI Project report. 2013. Available: gini-research.org/system/uploads/449/original/Japan.pdf?1370090592.

[48] Freixas X, Parigi BM, Rochet JC. Systemic risk, interbank relations, and liquidity provision by the central bank. J Money Credit Bank. 2000; 32: 611–638. 10.2307/2601198

[49] Eisenberg L, Noe TH. Systemic risk in financial systems. Manag Sci. 2001; 47: 236–249. 10.1287/mnsc.47.2.236.9835

[50] Haldane AG, May RM. Systemic risk in banking ecosystems. Nature. 2011; 469: 351–355. 10.1038/nature09659 21248842

[51] Barabási A L, Albert R. Emergence of scaling in random networks. Science. 1999; 286: 509–512. 10.1126/science.286.5439.509 10521342

[52] Golosovsky M, Solomon S. Stochastic dynamical model of a growing citation network based on a self-exciting point process. Phys Rev Lett. 2012; 109: 098701 10.1103/PhysRevLett.109.098701 23002894

[53] Alesina A, Rodrik D. Distributive politics and economic growth. National bureau of economic research. 1991; Working paper No. w3668.

[54] Levine R. Stock markets, growth, and tax policy. J Financ. 1991; 46: 1445–1465. 10.1111/j.1540-6261.1991.tb04625.x

[55] Ostry JD, Berg A, Tsangarides CG. Redistribution, inequality, and growth. 2014. International Monetary Fund working paper. Available: www.imf.org/external/pubs/ft/sdn/2014/sdn1402.pdf.

[56] Levine R, Zervos S. Stock markets, banks, and economic growth. Am Econ Rev. 1998; 88: 537–558.

[57] DeNavas-Walt C, Proctor B. Income and poverty in the United States: 2013. U.S Census Bureau working paper. Available: www.census.gov/content/dam/Census/library/publications/2014/demo/p60-249.pdf.

