# Correction: Modeling the Origin and Possible Control of the Wealth Inequality Surge

**DOI:** 10.1371/journal.pone.0135548

**Published:** 2015-08-07

**Authors:** The PLOS ONE Staff

In Fig 2, the fourth bar in Panel D is incorrectly stretched due to an error that occurred during the typesetting process. The publisher apologizes for the error. The correct version of Fig 2 can be seen here.

**Fig 2:**
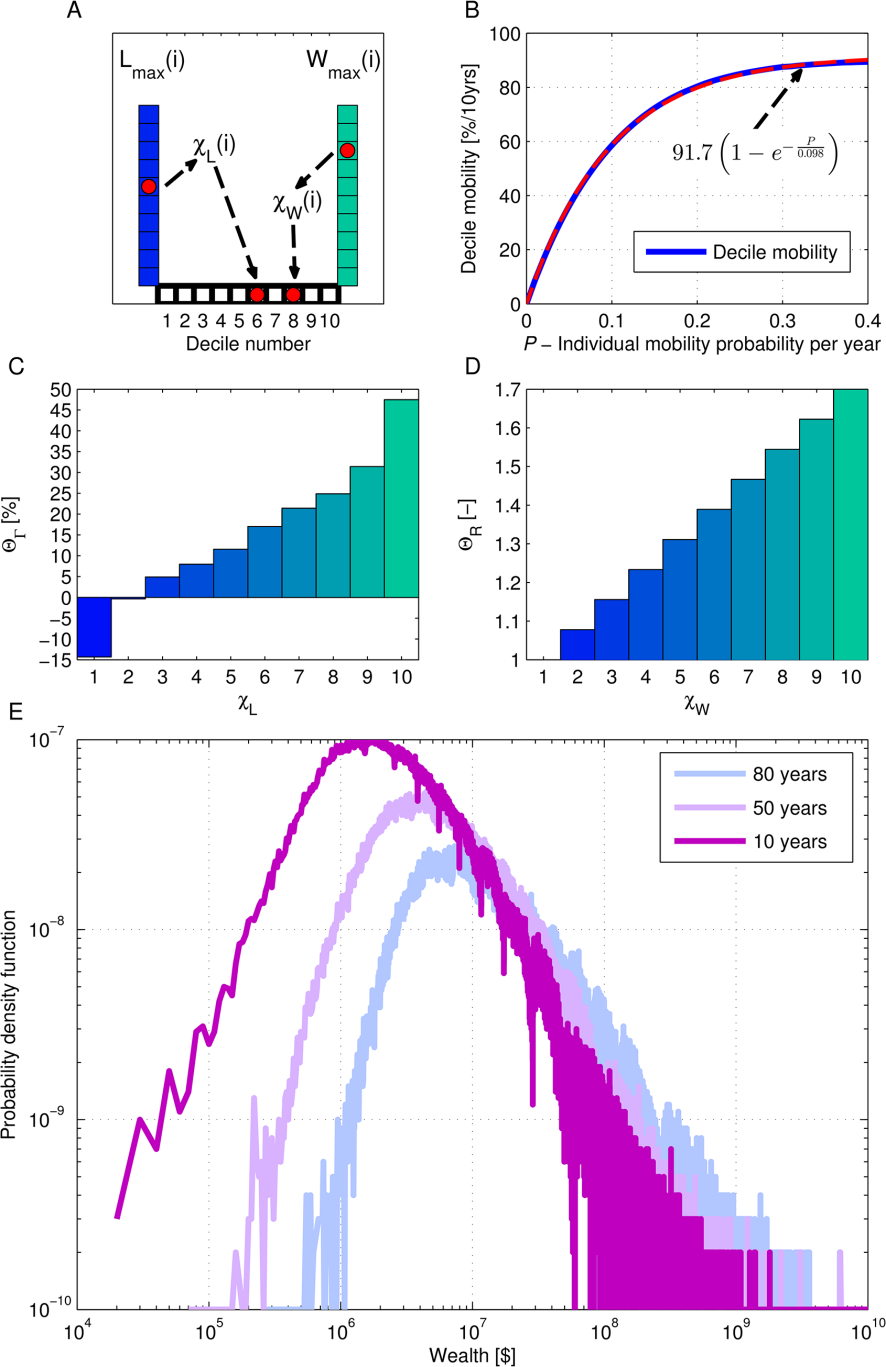
The characteristic behavior of the stochastic model. A: An illustration of the division to deciles—every time step *i*, each individual, with labor income *L* and wealth *W* is attached to a certain decile in labor income (*χ*_*L*_) and wealth (*χ*_*W*_) according to the distribution of the entire population; B: The decile mobility for 10 years is calculated following equation Eq (2) with *P* ranging from 0 to 0.4 per year. The decile mobility in the US was 75%–80% for 10-year periods during the past 50 years [44], leading to *P* values of 0.15–0.2 per year. The dashed red curve demonstrates that the decile mobility exponentially increases with *P*; C: The dependence of Θ_Γ_ on the labor income decile [41–43]; D: The dependence of Θ_*R*_ on the wealth decile; E: The model results for the distribution of wealth in the United States. The results were calculated for the historical values of the model parameters in the period 1930–2010, after 10 years (purple), 50 years (pink) and 80 years (light blue).
