# Supplementary figures and images for: Modeling the Origin and Possible Control of the Wealth Inequality Surge
Source: PLoS One. 2015 Jun 24;10(6):e0130181. doi: 10.1371/journal.pone.0130181 (PMC4479378; doi:10.1371/journal.pone.0130181)

A

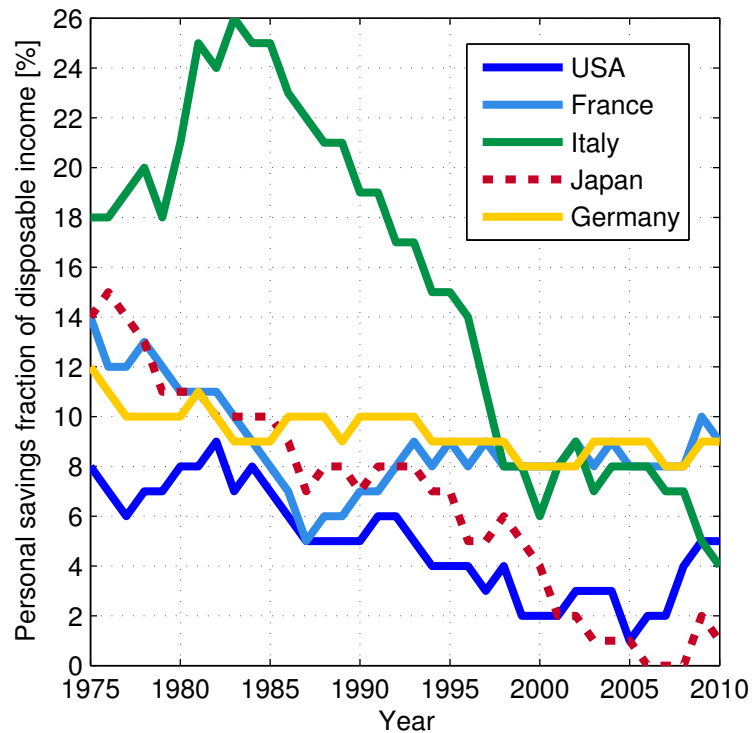

B

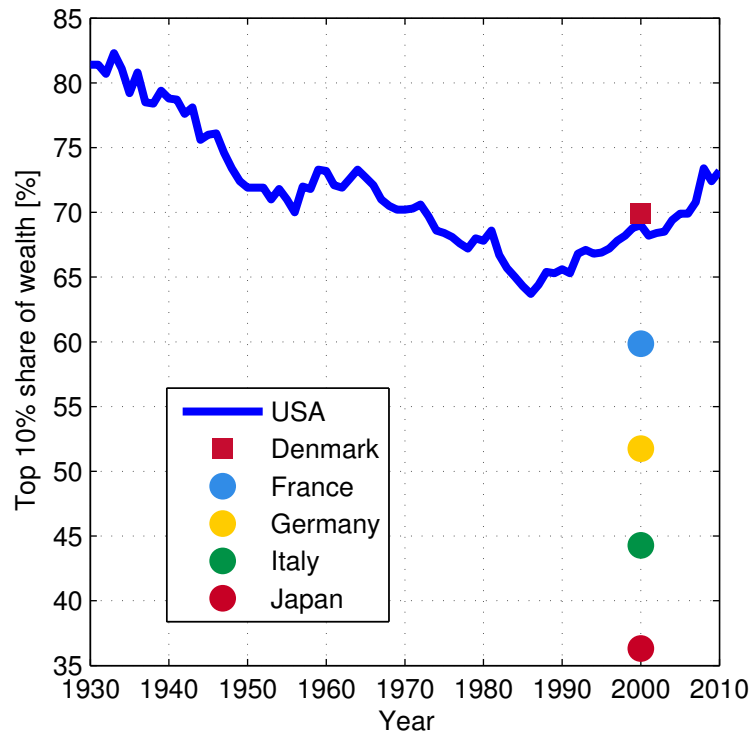

Supplement: S1 Fig — A: The private savings fraction is presented for the United States (blue), France (light blue), Germany (yellow), Italy (green) and Japan (dashed red) for 1975–2010. The data is taken from [23]; B: The top 10% share of wealth is presented for the United States (blue curve) for 1930–2010, as well as for Denmark (red square), France (light blue circle), Germany (yellow circle), Italy (green circle) and Japan (red circle) in 2000. The data is taken from [40]. This figure displays once again the link between private savings and wealth inequality. In addition, it shows that low income inequality does not necessarily imply low wealth inequality, as demonstrated by Denmark. In Denmark, the income inequality is one of the lowest in the world, while the level of wealth inequality is one of the highest in the world [39, 40]. (PDF) [file pone.0130181.s001.pdf]

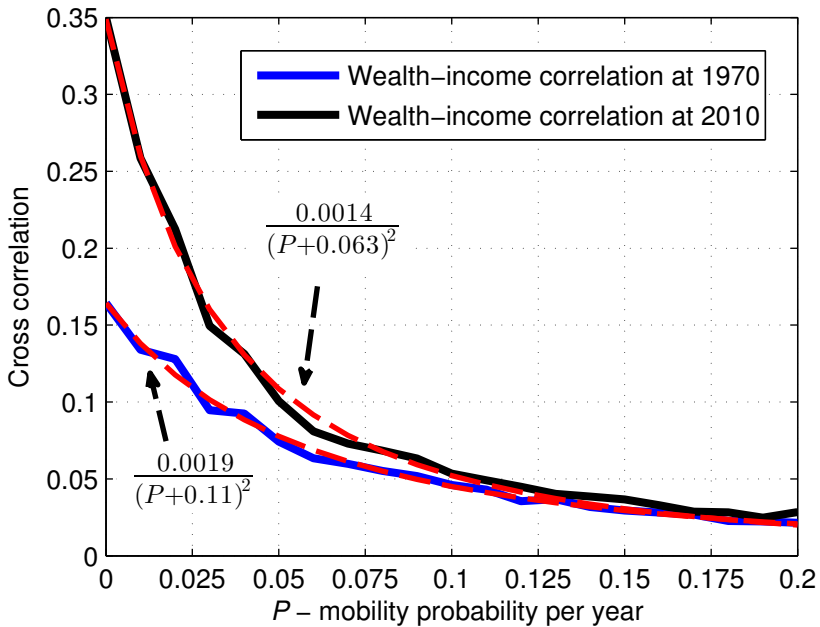

Supplement: S2 Fig — The correlation is presented for 1970 (blue) and for 2010 (black) as a function of the mobility probability P. The dashed red curves demonstrate that the correlation is inversely proportional to the square of the mobility probability. The figure demonstrates that the sensitivity of this correlation to the value of P becomes very low at about P = 0.1. (PDF) [file pone.0130181.s002.pdf]

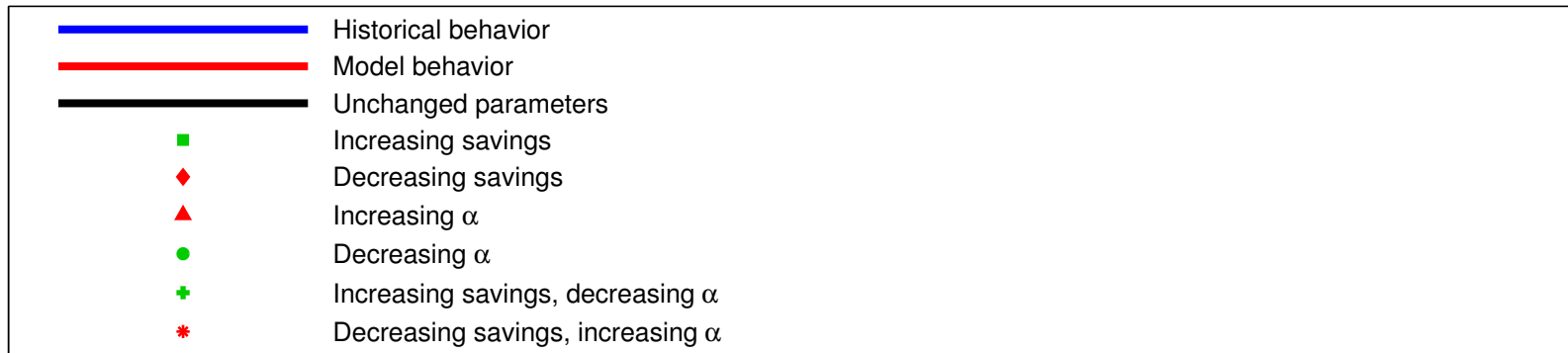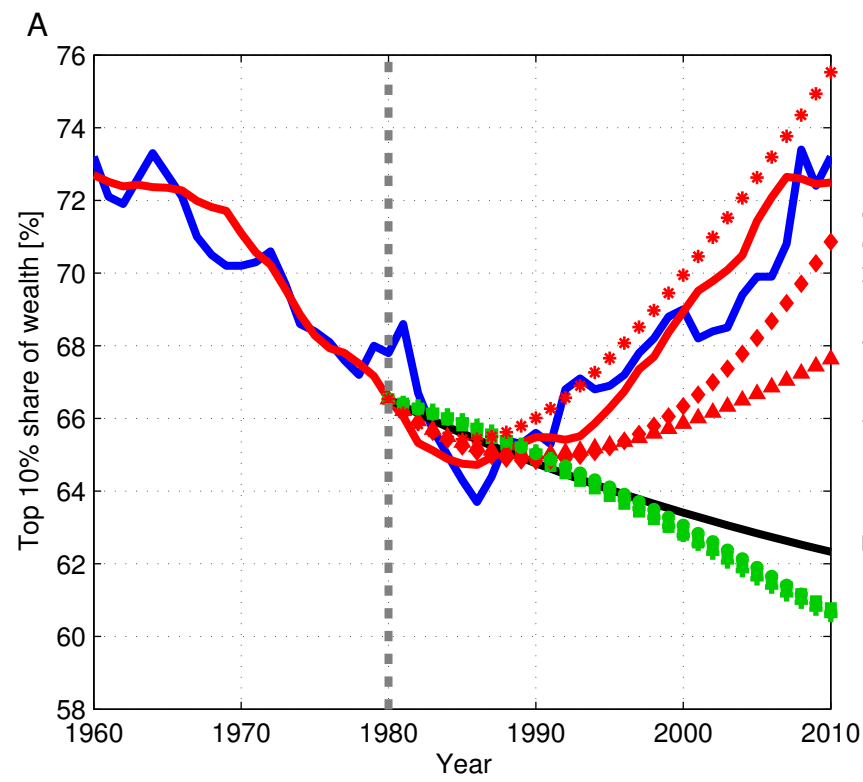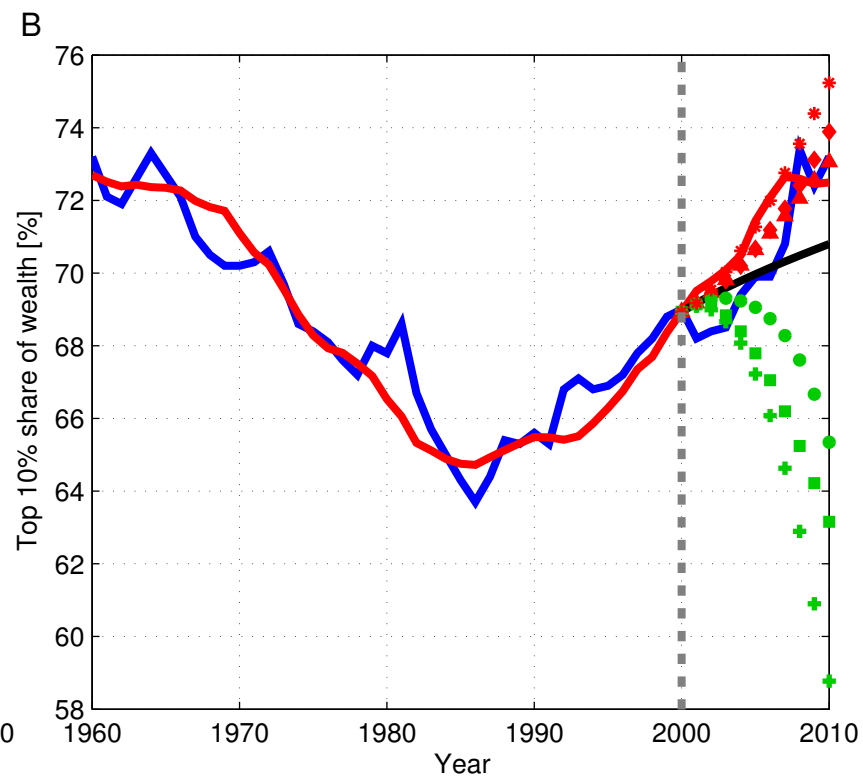

Supplement: S3 Fig — The blue and red curves present the historical market behavior and the model behavior for the historical values of the parameters, respectively (see Fig 4). The results for the various scenarios during 1980–2010 (A) and during 2000–2010 (B) are also presented: Unchanged parameter scenario (solid black curve), increasing savings scenario (green squares), decreasing savings scenario (red diamonds), increasing α scenario (red triangles), decreasing savings scenario (green circles), increasing savings and decreasing α scenario (green crosses) and decreasing savings and increasing α scenario (red stars). The dotted gray line separates the calculation using historical parameter values and the retrospective prediction. (PDF) [file pone.0130181.s003.pdf]

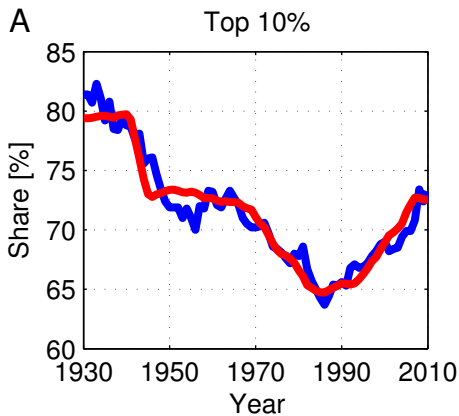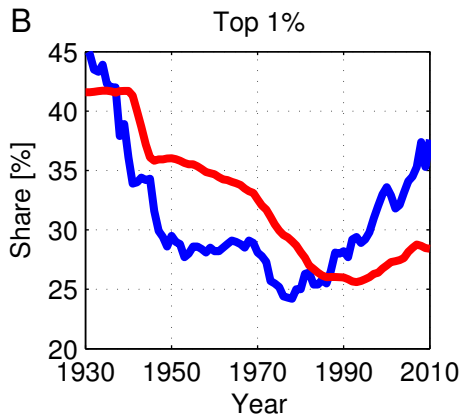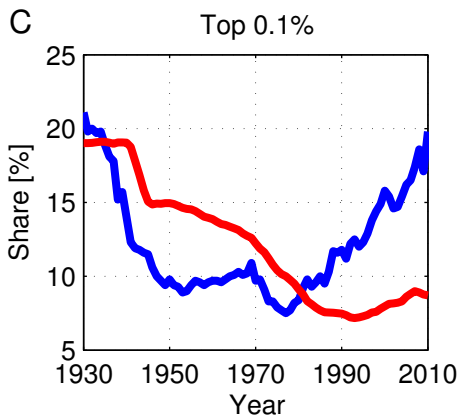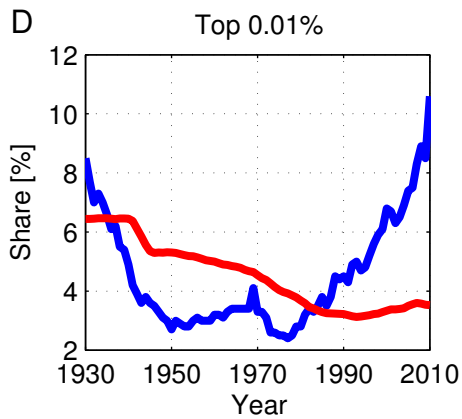

Supplement: S4 Fig — A-D: The historical share of wealth (blue) and the model results (red) owned by the top 10% of the population (A), the top 1% (B), the top 0.1% (C) and the top 0.01% (D). The historical data was taken from Saez and Zucman [22]. The results imply that different mechanisms govern the accumulation of wealth within the top fractiles of the population compared to within the vast majority of the population. Among the top fractiles, capital income is dominant and the fraction α becomes effectively much larger than 50%, which substantially change the dynamics of wealth accumulation. Moreover, the positive correlation between wealth and return on wealth [1, 11, 45] naturally contribute to a growing gap within the top 10%, 1% or 0.1%. We also note that the discrepancy between the model results and the data might be contributed to inaccurate data. Due to the smaller values, the data are more sensitive to errors. In addition, there exists a debate regarding the data provided by Saez and Zucman [22], which are inconsistent with other sources of wealth data [7]. (PDF) [file pone.0130181.s004.pdf]
